# Editorial: “Toward Precision Medicine in Vasculitis”

**DOI:** 10.3389/fimmu.2020.616221

**Published:** 2020-11-17

**Authors:** Federico Alberici, Giacomo Emmi, Andreas Kronbichler

**Affiliations:** ^1^Department of Medical and Surgical Specialties, Radiological Sciences and Public Health, University of Brescia, Brescia, Italy; ^2^Nephrology Unit, Spedali Civili Hospital, ASST Spedali Civili di Brescia, Brescia, Italy; ^3^Department of Experimental and Clinical Medicine, University of Firenze, Firenze, Italy; ^4^Internal Interdisciplinary Medicine Unit, Careggi University Hospital of Firenze, Firenze, Italy; ^5^Department of Internal Medicine IV (Nephrology and Hypertension), Medical University Innsbruck, Innsbruck, Austria

**Keywords:** antineutrophil cytoplasmic antibodies (ANCA)-associated vasculitis, biomarkers, Behçet’s syndrome, uveitis, treatment

Vasculitis is a group of rare systemic autoimmune diseases that may be classified according to the size of the vessels involved (1). The prognosis of these diseases has been dramatically improved by immunosuppression although toxicity of such therapies is not negligible and the response to treatment may vary exposing subgroups of patients to the risk of relapsing and refractory disease (2). Of note, the therapeutic arrays are rapidly expanding and new treatment protocols combining different target therapies are being proposed urging the identification of clinical characteristics as well as biomarkers able to identify subgroups of patients more likely to benefit from specific approaches (3). Furthermore, the deepening of the understanding of the mechanisms of action of the drugs employed poses also the rationale for patients’ monitoring that may in some cases guide re-treatment. For example, in the field of antineutrophil cytoplasmic antibodies (ANCA) associated vasculitis (AAV), the kinetics of CD20+ B-cells repopulation or increase of the ANCA titer in patients treated with the chimeric monoclonal anti-CD20 antibody rituximab, may be associated to an increased relapse risk (4). Moreover, these biomarkers have been explored as potentially able to guide patients’ re-treatment (5). In this perspective, the research is also proposing biomarkers able to identify subgroups of patients less likely to respond to a specific therapy as well as posing the rationale for combining different biological drugs (6).

The aim of this special issue is to describe the state of the art of precision medicine in vasculitis. This collection contains 14 articles including eight original research publications and six reviews. The nice balance between original manuscripts and literature reviews supports the idea of this topic as an evolving concept.

Two reviews approached the broad topic of how genetic association and pharmacogenetics studies as well as studies of single-gene high-penetrance mutations, epigenetics factors, metabolomics and proteomics are contributing to the precision medicine in this field. These approaches are providing the rationale for advanced diseases classification, patients stratification while improving the understanding of the pathogenesis as well as identifying new therapeutic targets (Acosta-Herrera et al.; Demirkaya et al.).

Of note, the vast majority of the remaining manuscripts of this issue focused on small vessels vasculitis: six original papers and three reviews.

In the three reviews of the literature, key opinion leaders described the state of the art in the field of precision medicine in AAV. Wallace et al. focused on the role of ANCA specificity in defining a personalized approach to patients’ management further supporting the superiority for an ANCA-based classification of AAV compared to a classification based on clinical characteristics (Wallace and Stone). Shochet et al. focused on the role of animal models of AAV in the field of translational research addressing the intriguing issue of difficulties in the generation of animal models for PR3-ANCA AAV, leaving uncertainties on the pathogenetic role of PR3-ANCA compared to the well-established pathogenetic role of MPO-ANCA. Segelmark et al. reviewed the rationale and the data supporting the potential role of IdeS and EndoS, two enzymes produced by *Streptococcus pyogenes* capable of degrading IgG, and their potential as innovative therapeutic strategies in antibody mediated diseases such as small vessels vasculitis.

Original articles on AAV published in this issue focused on insights into disease pathogenesis, potential biomarkers, improvement in phenotypic characterization, and identification of potential therapeutic targets. Sun et al. demonstrated that thrombin could enhance MPO-ANCA induced activation of glomerular endothelial cells and that the protein sphingosine-1-phospahte (S1P) may act as link of the hyper-activation of the coagulation and inflammation system. This paper therefore provides a further rationale to the well-known tight link between inflammation and thrombosis as well as new potential therapeutic targets.

Two works focused on potential new biomarkers. Mhaonaigh et al. showed that low-density granulocytes may be associated to active vasculitis (Ui Mhaonaigh et al.), while Van Daalen et al. focused on the study of podocytes in patients with kidney involvement of AAV. In this report, of interest, proteinuria ten weeks after diagnosis correlated with podocytes foot process width; moreover, this characteristic was associated to different histological features at light microscopy. This study would be in support for a thorough assessment of podocytes at the moment of kidney biopsy providing important prognostic information further supporting the central role of kidney biopsies in patients with AAV (van Daalen et al.).

In everyday clinical practice, relying on clear and validated phenotypic classification of patients is key in order to have prognostic information especially in the field of rare diseases. Marques et al. described a big multicenter French cohort of patients with anti-glomerular basement membrane (anti-GBM) disease identifying several factors associated to poor prognosis in terms of overall survival as well as risk of end-stage kidney disease contributing significantly to the improvement of patients stratification (Marques et al.).

Eventually, the aim of focusing on precision medicine is improving patients’ management, which also includes the identification of new potential therapeutic targets: two of the articles on AAV focused on this aspect. Pang et al., *via* the study of recombinant PR3 antigens and their interaction with the monoclonal antibody moANCA518, hypothesizes that the interaction between PR3 and PR3-ANCA may represent a potential target of interest (Pang et al.). On the other hand, Werner et al. showed that the negative co-stimulator B- and T-lymphocyte attenuator (BTLA) was diminished on double negative T-cells in remission samples of AAV patients and correlated with disease activity and relapse rate. The same study showed that T-cell inhibition *via* BTLA during T-cell receptor-mediated stimulation led to suppression of T-cell proliferation, inhibition of interleukin (IL)-17 and interferon (IFN)-gamma, suggesting that this may also represent a therapeutic target.

Behçet syndrome (BS) is a systemic vasculitis frequently posing diagnostic challenges and for which classification uncertainties do exist; a review by Bettiol et al. report the phenotypic rationale for sub-classifying BS in three subgroups (mucocutaneous and articular; extra-parenchymal neurological and peripheral vascular phenotype; the parenchymal neurological and ocular phenotype), further supporting the clinical observation that this condition may indeed be a complex spectrum of diseases ranging from mild phenotypes to life/organ threatening forms. Of note, the same article recommends a different therapeutic management for these phenotypes with a huge impact on patients’ management.

In an original manuscript on BS, Emmi et al. focused on circulating hematopoietic progenitor cells (CPC), identifying a reduction of this stem cells in cases compared to healthy controls; moreover, the data would support the hypothesis that oxidative stress may contribute to CPC apoptosis suggesting a possible role for these mechanisms in counteracting the vascular repair actions of these cells (19).

Typically, BS is characterized by ocular involvement (i.e posterior uveitis/panuveitis). Bonacini et al. profiled cytokines in aqueous humor of patients with non-infectious uveitis secondary to BS, Vogt Koyanagi Harada disease and healthy controls. The authors found a different intra-ocular cytokine profile in patients with auto-inflammatory uveitis. At the same time, the profile of cytokines identified as potential therapeutic targets are already part of the therapeutic armamentarium and their impact should be better clarified in future.

In conclusion, this special issue collects reports highlighting current state of the art in the field of precision medicine in vasculitides as well as original manuscripts identifying new biological as well as phenotypic markers that may contribute to further progress in this field. We believe that the reader will benefit from a broad overview that will contribute deeply to the general the understanding on this topic.

## Author Contributions

FA drafted a version. GE and AK reviewed and completed it. All authors contributed to the article and approved the submitted version.

## Conflict of Interest

The authors declare that the research was conducted in the absence of any commercial or financial relationships that could be construed as a potential conflict of interest.
